# M48U1 and Tenofovir combination synergistically inhibits HIV infection in activated PBMCs and human cervicovaginal histocultures

**DOI:** 10.1038/srep41018

**Published:** 2017-02-01

**Authors:** Giuseppina Musumeci, Isabella Bon, David Lembo, Valeria Cagno, Maria Carla Re, Caterina Signoretto, Erica Diani, Lucia Lopalco, Claudia Pastori, Loïc Martin, Gilles Ponchel, Davide Gibellini, Kawthar Bouchemal

**Affiliations:** 1 Department of Experimental, Diagnostics and Specialty Medicine (DIMES), Microbiology Section, University of Bologna, 40138 Bologna, Italy; 2 Department of Clinical and Biological Sciences, University of Torino, 10043 Orbassano, Italy; 3 Department of Diagnostics and Public Health, Microbiology and Virology Unit, University of Verona, Strada Le Grazie 8, 37134 Verona, Italy; 4 Division of Immunology, Transplantation and Infectious Diseases, San Raffaele Scientific Institute, 20132 Milan, Italy; 5 CEA, iBiTecS, Service d’Ingénierie Moléculaire des Protéines (SIMOPRO), Gif sur Yvette, F-91191, France; 6 Institut Galien Paris Sud, CNRS UMR 8612, Univ. Paris-Sud, Université Paris Saclay, 5 rue J-B. Clément, 92296, Châtenay-Malabry cedex, France

## Abstract

Microbicides are considered a promising strategy for preventing human immunodeficiency virus (HIV-1) transmission and disease. In this report, we first analyzed the antiviral activity of the miniCD4 M48U1 peptide formulated in hydroxyethylcellulose (HEC) hydrogel in activated peripheral blood mononuclear cells (PBMCs) infected with R5- and X4–tropic HIV-1 strains. The results demonstrate that M48U1 prevented infection by several HIV-1 strains including laboratory strains, and HIV-1 subtype B and C strains isolated from the activated PBMCs of patients. M48U1 also inhibited infection by two HIV-1 transmitted/founder infectious molecular clones (pREJO.c/2864 and pTHRO.c/2626). In addition, M48U1 was administered in association with tenofovir, and these two antiretroviral drugs synergistically inhibited HIV-1 infection. In the next series of experiments, we tested M48U1 alone or in combination with tenofovir in HEC hydrogel with an organ-like structure mimicking human cervicovaginal tissue. We demonstrated a strong antiviral effect in absence of significant tissue toxicity. Together, these results indicate that co-treatment with M48U1 plus tenofovir is an effective antiviral strategy that may be used as a new topical microbicide to prevent HIV-1 transmission.

According to World Health Organization (WHO)^1^ approximately 37 million people worldwide are currently infected with HIV-1, with 2 million new cases of infection and 1.2 million deaths from AIDS-related causes per year. Almost 50% of infections worldwide are in women, and the majority of detectable infections occur in sub-Saharan Africa^1^,^2^, where 56% of the regional cases are in women. In addition, AIDS is the leading cause of death in women of reproductive age in this region^1^,^3^. These data indicate the need to develop useful approaches to protect women from HIV-1 infection, especially since sexual intercourse is the principal route of HIV-1 transmission^1^. Indeed, some reports have indicated that approximately 85% of HIV-1 infections are transmitted by sexual intercourse^4^,^5^, and heterosexual contact is heavily involved in the spread of HIV-1. The use of combined antiretroviral therapy (cART) cannot eradicate HIV-1 infection in an individual, however cART counteracts the development of disease and dramatically increases the life expectancy^6^,^7^. Furthermore, the suppression of HIV-1 replication by cART leads to a decrease in the viral load in a treated HIV-1 positive patient, which reduces the risk of HIV-1 transmission^8^. Unfortunately, all vaccine strategies that have been evaluated to date have failed to induce effective immunological protection from HIV-1 because of its genetic variability and cellular latency of HIV-1^9^.

Behavioural approaches (i.e., male circumcision, condom use) have been used for preventing HIV-1 transmission, but these methods are inefficient at reducing sexual transmission of HIV-1, particularly in sub-Saharan Africa, where one of the highest risk factors for acquiring HIV-1 in women is low condom use by the male partner^2^,^10^. A promising strategy for inhibiting HIV-1 transmission is represented in microbicides^11^,^12^. Some mathematical models have demonstrated that the proper use of microbicides may decrease the number of infected women by interfering with the spread of HIV-1 infection^13^,^14^. In HIV-1 treatment, microbicides are drugs with antiretroviral properties, which are applied topically in the form of a vehicle gel or film to either the vaginal or rectal mucosa pericoitally^11^,^12^. The recent preparation of microbicides with long-term release from a vaginal ring could represent a valid form of pre-exposure prophylaxis^15^. It has been recently proposed that the use of specific drugs taken *per os* inhibits HIV-1 transmission increasing treatment compliance^16^,^17^,^18^. Early approaches to microbicide application were developed using polyanionic gels and surfactant-based products applied in vaginal and rectal cell models, but were not reliable in preventing HIV-1 infection^19^. Furthermore, some trials using HIV-1 attachment inhibitors, including PRO 2000, cellulose sulfate and Carraguard, failed to show anti-HIV-1 activity when these molecules were used as microbicides^20^,^21^,^22^,^23^. The first encouraging data on the use of microbicides to prevent transmission were obtained in the CAPRISA 004 study, during which a gel with tenofovir was applied to the vaginal mucosa and reduced the risk of acquiring HIV-1. The patients treated with tenofovir gel were 39% less likely to become infected with HIV-1 compared to the control group^24^. Similarly, pre-exposure chemoprophylaxis using oral administration of Truvada, a pharmacological preparation containing tenofovir, disoproxil fumarate and emtricitabine, resulted in a 44% reduction in the incidence of rectal transmission of HIV-1 among men who have sex with men^25^. Unfortunately, other studies carried out in cohorts of women treated with tenofovir gel treatment (the VOICE and the FACTS 001 trials) or oral Truvada (the FEM PrEp study) did not confirm these initial results, due to low patient adherence to the treatment^21^,^26^.

Several new antiretroviral microbicides have been developed and shown to interfere with specific phases of the HIV-1 replication cycle. In particular, the most promising targets are those that inhibit HIV-1 entry and reverse transcription. HIV-1 entry inhibitors are considered valuable tools as microbicides, and specifically promising agents are those directed against the interaction of cellular CD4 and the HIV-1 gp120 protein since protection would be independent of co-receptor tropism. Previous studies with BMS-378806, dendrimers, CADA and miniCD4 mimetic peptides demonstrated their strong inhibitory effects on HIV-1 infection of histocultures *in vitro* and/or during studies using vaginal challenge of non-human primates with the SHIV-1_162P3_ strain, suggesting the potential use of these molecules as microbicides^27^,^28^,^29^,^30^,^31^. Recently, treatment regimens composed of two or more drugs that target different phases of the HIV-1 replication cycle have been considered to increase the overall antiviral effects of microbicides during HIV-1 transmission^32^,^33^. MiniCD4 M48U1 is a peptide that inhibits HIV-1 infection^34^ through the binding with gp120, with a higher affinity (100-fold titer binding) than that of gp120 binding soluble CD4, due to a higher rate of association and slow rate of dissociation^34^,^35^. This drug may be a good candidate as a microbicide because previous observations in a simian model demonstrated that M48U1 formulated in hydroxyethylcellulose (HEC) and pluronic-based hydrogels inhibit HIV-1 infection^36^.

In this report, we studied the antiretroviral efficacy of miniCD4 M48U1 alone or in combination with tenofovir *in vitro* in both PBMCs and cervicovaginal histocultures. We propose that this combination may be used as a novel microbicide for inhibiting HIV-1 transmission through sexual intercourse.

## Methods

### Virus stocks and drugs

HIV-1 X 4 or R5 strains were used for the experiments. HIV-1_IIIb_ (subtype B, X4 tropism), HIV-1_ADA_ (subtype B, R5 tropism), HIV-1_ARV-2_ (subtype B, R5/X4 tropism), HIV-1_RU132_ (subtype G, R5 tropism), HIV-1_BaL_ (subtype B, R5 tropism), and HIV-1_CBL4_ (subtype D, X4 tropism) were obtained from NIBSC (NIBSC, London, UK). Three HIV-1 subtype B isolates (HIV-1_AMBR5N_, HIV-1_ARR5X4_, and HIV-1_CSBX4_) were obtained from naïve (HIV-1_AMBR5N_ R5 tropism) and cART-treated (HIV-1_ARR5X4_ R5/X4 tropism, HIV-1_CSBX4_ X4 tropism) HIV-1-positive subjects. Two HIV-1 subtype C isolates (HIV-1_FBR5N_ and HIV-1_SLR5N,_ both R5 tropism) were obtained from acutely infected naïve patients. For all the HIV-1 isolates, cellular receptor tropism was determined by classical genotyping method^37^. Stocks of reference HIV-1 strains were prepared in C8166 cells (HIV-1_IIIb_) or in activated PBMCs (HIV-1_ADA_, HIV-1_ARV-2_, HIV-1_RU132_, HIV-1_BaL_ and HIV-1_CBL4_)^38^. The primary viral isolates were obtained from HIV-1-infected subjects using a co-culture technique, as described previously^38^. PBMCs from HIV-1 infected patients were obtained after giving their written consent for study, according to the Helsinki declaration. Briefly, the HIV-1_IIIb_ stock was obtained by clarifying the supernatant of an HIV-1-infected C8166 culture free of bacteria contamination using low-speed centrifugation. The reference HIV-1 strains and primary isolates were obtained by culturing approximately 1 × 10^6^ PBMCs obtained from the buffy coat of donor HIV-1 seronegative blood. The cultured PBMCs were treated for 72 h with phytohaemagglutin (PHA; 5 μg/ml, Sigma, St.Louis, MO, USA) plus IL-2 (10U/ml; Pierce, Rockford, IL, USA). The cell culture supernatants, after centrifugation at 7,000 rpm for 15 min, were tested twice per week for the presence of HIV-1 p24 core protein. The viral stocks were titrated with an HIV-1 gag p24 antigen ELISA kit (Biomerieux, Marcy L’Etoile, France). Viral stocks were assessed at a concentration of 1000 ng p24/ml and used in the experiments at final concentration of 5 ng p24/ml.

The genomic sequence of each full-length transmitted/founder (T/F) HIV-1 strain was deduced using a mathematical model of HIV-1 sequence evolution during acute clinical infection and an experimental strategy based on single genome amplification (SGA) of plasma vRNA/cDNA, followed by direct sequencing of SGAs products without a cloning step^39^,^40^. The *bona fide* T/F infectious molecular clones (IMCs) including pTHRO.c/2626 and pREJO.c/2864, were described previously by Ochsenbauer and coworkers^41^, and John C. Kappes and C. Ochsenbauer contributed the T/F IMCs to the NIH ARRRP for public availability. The 293T cell-derived stocks of replication competent IMCs were generated by transfection of proviral DNA using FuGENE 6, according to the manufacturer’s protocol (Promega, Madison, WI, USA). Supernatants containing virus were harvested 72 hours post-transfection, clarified at 1,800 rpm for 20 min, and frozen at −70 °C. Four replicates of five-fold diluted supernatants containing of T/F IMCs were added to a 96-well flat-bottom plate containing 1 × 10^4^ TZM-bl cells per well in 10% D-MEM growth medium with 7.5 μg/mL of DEAE-dextran (Sigma) in a final volume of 200 μL. After 72 hours of incubation at 37 °C, 100 μL of culture medium were replaced with 100 μL of Bright-Glo luciferase reagent (Promega). After 2 minutes of incubation, 150 μL of the cell lysate were transferred to a 96-well white solid plate and luminescence was measured using a Victor Light 2030 luminometer (Perkin Elmer). Fifty per cent infectious dose (ID_50_) titres were defined as the reciprocal of the virus dilution yielding 50% positive wells (Reed-Muench calculation). 293T cells, TZM-bl cells and T/F IMCs were obtained through the NIH AIDS Research and Reference Reagent Program.

The M48U1 peptide^42^ was synthesized by Pepscan Presto Inc. (Lelystad, The Netherlands) using a solid phase peptide synthesis procedure. The M48U1 peptide contains a p-(cyclohexylmethyloxy)phenylalanine residue at aminoacid 23 able to enhance its affinity to the Phe43 cavity of gp120^34^,^35^, and was purified after refolding by reverse-phase high-performance liquid chromatography as described previously^42^. The sequence of M48U1 was previously published^35^. Tenofovir was obtained from NIBSC. HEC hydrogel was prepared as described in a previous report^43^. Briefly, the HEC hydrogel stock (1.5%) was prepared by adding 1.5 g of gelling polymer HEC (Natrosol 250 M Pharm, Aqualon, USA) to a vial containing citrate buffer (5 mM, pH 4.5). The final formulation was mixed with a mechanical stirrer until complete dissolution of the HEC powder. The M48U1 and/or tenofovir preparations were formulated by adding M48U1 and/or tenofovir to HEC hydrogel. Drug-containing hydrogels were then homogenized by magnetic mixing. The desired concentrations of drugs and HEC were obtained by performing appropriate dilutions of these molecules in RPMI 1640 medium (Gibco, Gaithersburg, MD, USA) immediately before their use in the experiments.

### Cell isolation and activation

Peripheral blood samples were collected from healthy blood donors during routine laboratory analyses at the Blood Bank of S. Orsola-Malpighi Hospital in Bologna according to the rules established by Italian law (Legislative Decree 03-03-2005, published in G.U. n. 85, 13.04.2005). No approval from the Ethics Committee was requested because all blood samples were obtained from anonymous donors with no identifying information. A Ficoll gradient (Ficoll-Histopaque, Pharmacia, Uppsala, Sweden) was used to separate the PBMCs from the blood. PBMCs were seeded in RPMI 1640 plus 10% FCS and 2 mM L-glutamine at 5 × 10^5^ cells/ml. The PBMCs were activated by treatment with PHA (5 μg/ml; Sigma) plus IL-2 (10 U/ml; Pierce) treatment for three days. The culture medium was replaced every three days with fresh medium (RPMI 1640 plus 10% FCS, 2 mM L-glutamine and 10 U/mL IL-2).

### HIV-1 infection and antiviral treatment

The HIV-1_BaL_ and HIV-1_IIIb_ laboratory strains (5 ng/ml of HIV-1 gag p24) were pre-incubated for one hour at 37 °C with increasing concentrations of either M48U1 (0, 0.0033, 0.033, 0.33, 1 or 3.3 μM) formulated in 0.25% HEC or tenofovir (0, 0.5, 1.25, 2.5, 5 or 10 μM) formulated in 0.25% HEC. Then, the solutions were added to activated PBMCs that were adjusted to a final density of 1 × 10^6^ cells/ml for a two hour incubation at 37 °C. After four washes in PBS, the cells were seeded at 5 × 10^5^ PBMCs/ml in culture plates containing fresh medium with various concentrations of the M48U1 or tenofovir formulations. The HIV-1 gag p24 content was determined at seven days post-infection in the culture supernatant using the HIV-1 p24 antigen ELISA kit (Biomerieux). Mock-infected PBMCs were used as the negative control. The experiments using other laboratory strains and viral isolates were performed as indicated above with a single concentration of M48U1 (1 μM) or tenofovir (5 μM) formulated in 0.25% HEC. The viability of the activated PBMCs was evaluated by the Trypan Blue exclusion technique in the presence of various concentrations of M48U1 or tenofovir formulated in 0.25% HEC using the protocol described above.

To analyze the inhibition of infection with HIV-1 T/F strains, a final concentration of 1 μM or 6.7 μM of M48U1 and 5 μM or 20 μM of tenofovir both in 0.25% HEC, were plated in triplicate in a 96-well flat bottom plate in 10% D-MEM containing 20 TCID_50_ of each T/F IMC (150 μL/well), and incubated for 1 hour at 37 °C. TZM-bl cells (1 × 10^4^/well in a 100 μL volume) in 10% D-MEM growth medium containing DEAE-dextran (Sigma) at a final concentration of 7.5 μg/mL were then added to the plate. Assay controls included replicate wells of TZM-bl cells alone in HEC 0.25% (cell control) and TZM-bl cells with virus (virus control). Following 72 hours of incubation at 37 °C, 150 μL of culture medium were removed from each well and replaced with 100 μL of Bright-Glo luciferase reagent (Promega). After 2-minutes of incubation, 150 μL of the cell lysate were transferred to a 96-well white solid plate and luminescence was measured using a Victor Light 2030 luminometer (Perkin Elmer). The percentage of infection for each T/F strain was calculated by comparing the relative luminescence units (RLU) of the virus control wells after subtraction of the cell control RLU^44^.

### Drug combination analysis

Different concentrations of tenofovir (0, 0.1, 0.5, 1.25, 2.5, 5 or 10 μM) and M48U1 (0, 0.001, 0.005, 0.03, 0.15, 0.75 or 1.5 μM) were co-formulated in 0.25% HEC and tested in activated PBMCs challenged with HIV-1_BaL_ (5 ng/ml of HIV-1 gag p24) using the protocol described above. The HIV-1 gag p24 content was determined at seven days post-infection in the culture supernatants using the HIV-1 p24 antigen ELISA kit (Biomerieux). The data were analyzed using an isobologram according to previously reported methods^45^,^46^,^47^. In brief, IC_50_ values were used to calculate the fractional inhibitory concentration (FIC) as follows: FICx (EC_50_ of compound “X” in combination/IC_50_ of compound alone); FICy (IC_50_ of compound “Y” in combination/IC_50_ of compound alone). When the FIC index, which corresponds to the sum of the FIC values of the combined compounds (FIC index = FICx + FICy), is equal to 1, the combination is additive; when the FIC index is between 1.0 and 0.5, the combination is partially synergistic; when it is < 0.5, the combination is synergistic; when it is between 1.0 and 2.0, the combination is partially antagonistic; and finally, when it is > 2.0, the combination is antagonistic. The combinations of drug concentrations M48U1/tenofovir were: 0/10 μM; 0.001/5 μM; 0.005/2.5 μM; 0.03/1.25 μM; 0.15/0.5 μM; 0.75/0.1 μM and 1.5 /0 mm. Data from drug combination studies were also evaluated using the MacSynergy II analysis program for multiple drug interactions^48^.

### Analysis of the effects of M48U1 and/or tenofovir formulated in 0.25% HEC in the HIV-1-infected cervicovaginal histoculture model

The EpiVaginal Tissue Model (VLC-100FT; MatTek Corporation, Ashland, MA, USA) was maintained in a 24-well plate according to the manufacturer’s instructions. This *in vitro*-reconstituted, full thickness vaginal tissue model was formed using a complete, stratified vaginal–ectocervical epithelial layer with Langerhans cells and an additional lamina propria containing fibroblasts. On the apical surface of each tissue-containing well, we added 6.7 or 0.67 μM M48U1 in 0.25% HEC and 2 or 20 μM tenofovir in 0.25% HEC. We used two different mixtures co-formulated in 0.25% HEC: i) 0.67 μM M48U1 plus 2 μM tenofovir and ii) 6.7 μM M48U1 plus 20 μM tenofovir. Subsequently, the wells were challenged with HIV-1_BaL_ (25 ng p24/tissue well) for 24 hours. Mock-infected 0.25% HEC-treated wells were used as negative control. The apical surface of each tissue was washed twice with PBS after incubation with HIV-1_BaL_ and the same formulations were added on day 1 for 24 hours. The underlying media was changed every day, and cervicovaginal tissues were harvested on day 4 post-infection.

Total DNA was extracted and purified from the tissues using the QIAamp DNA Mini Kit (Qiagen, Hilden, Germany). Proviral DNA was quantified by real-time quantitative PCR assay using the HIV-1 DNA qPCR kit (Diatheva, Fano, Italy) based on SYBR Green chemistry as indicated by the manufacturer. Briefly, the amplification was carried out in 20 μL reactions consisting of 10 μL of HIV-1 DNA 2X master mix (Diatheva), which contained 0.5 μL of Hot-Rescue PLUS DNA polymerase (Diatheva) plus long terminal repeat (LTR) region-specific oligos (forward primer: 5′-TAGCAGTGGCGCCCGA-3′ and reverse primer 5′-TCTCTCTCCTTCTAGCCTCCGC-3′ designed to amplify a 161 bp fragment^49^ derived from the 5′-LTR U5 end to the Gag-Pol start site of the sequence of HIV-1) and 200 ng of DNA. Total DNA amount was determined by spectrophotometry. The amplification was carried out as follows: an initial cycle of denaturation step at 95 °C for 15 minutes, followed by 10 cycles of denaturation at 95 °C for 15 seconds and annealing/extension at 68 °C for 35 seconds, and a final 35 cycles of denaturation at 95 °C for 15 seconds and annealing/extension with fluorescence acquisition at 68 °C for 35 seconds. At the end of the amplification cycles, a quantitative analysis was performed by interpolation of the data obtained using a standard curve. The data were normalized to those of a parallel amplification of the β-globin gene as described previously^31^. The manufacturer specified that the limit of detection of the assay was three viral copies/500 ng total DNA.

### Analysis of the irritation potential of M48U1 and tenofovir in the EpiVaginal system

The cytotoxicity of M48U1, tenofovir and both in combination formulated in 0.25% HEC hydrogel was analysed with an MTT ET-50 tissue viability assay, followed by determining the level of lactate dehydrogenase (LDH). We tested the irritation potential of 6.7 μM M48U1 alone, 20 μM tenofovir alone and 6.7 μM M48U1 plus 20 μM tenofovir formulated in 0.25% HEC hydrogel following administration to the apical surface of cervicovaginal cultures and incubation for 1, 4, 18 or 96 hours. At the end of the incubation at 37 °C, the hydrogel was discarded and the cervicovaginal cultures were washed with PBS to remove any residual hydrogel. The cervicovaginal cultures were analyzed using the MTT kit protocol (MatTek Corporation). Cell cultures incubated with sterile distilled water were employed as a negative control. Triton X-100 (1%)-treated cultures were used as the positive control. The LDH cytotoxicity detection kit (TaKaRa Bio Inc, Japan) was used to detect LDH release from the tissues according to the manufacturer’s protocols. The inflammatory response was evaluated by measuring the level of cytokine IL-1α released into the culture medium of EpiVaginal tissues treated with tenofovir and M48U1 for 1, 4, 18 or 96 hours, as previously reported^50^. After incubation, the concentration of IL-1α in the culture medium was measured using the IL-1α ELISA Kit, according to the manufacturer’s instructions (Bender Medsystem, Wien, Austria). The concentration of IL-1α was calculated by interpolation from a standard calibration curve.

### Statistical analyses

The results were expressed as the mean ± standard deviations (SD) of three separate experiments performed in duplicate or triplicate. Non-parametric Mann Whitney, one-way ANOVA and two-way ANOVA followed by Bonferroni test were used for the statistical comparisons. The IC_50_ and 95% CI values were calculated using the Prism (GraphPad Software, San Diego, CA) and ic50tk (www.ic50.tk) software.

## Results

### Infection of activated PBMCs with R5-tropic HIV-1_BaL_ or X4-tropic HIV-1_IIIb_ was inhibited by treatment with HEC formulations of miniCD4 M48U1 or tenofovir

**Figure 1:**
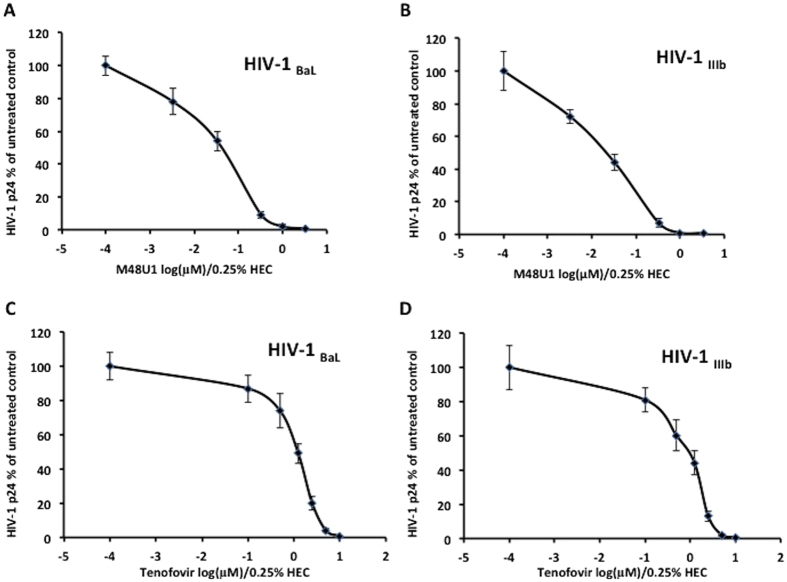
M48U1 and tenofovir formulated in 0.25% HEC each inhibited the replication of both R5-tropic HIV-1_BaL_ and X4-tropic HIV-1_IIIb_ in activated PBMCs. Either the R5-tropic HIV-1_BaL_ or X4-tropic HIV-1_IIIb_ (5 ng/ml of HIV-1 gag p24) strain was incubated for 1 hour at 37 °C in RPMI 1640 medium with increasing concentrations of M48U1 (**A**,**B**) or tenofovir (**C**,**D**) formulated in HEC at a final concentration of 0.25%. After this incubation, these solutions were used to resuspend activated PBMCs for 2 hours at 37 °C. HIV-1 replication was monitored by a HIV-1 p24 ELISA assay on the cell culture supernatants performed on day 7 post-infection. The data were expressed as the means ± standard deviations (±SD) of the level of HIV-1 gag p24 relative to untreated controls (set to 100%). Three experiments in triplicate were performed.

The antiviral activity of each drug, M48U1 and tenofovir, formulated in 0.25% HEC hydrogel was determined in an activated PBMC model. In the first set of experiments, we pre-incubated either R5-tropic HIV-1_BaL_ or X4-tropic HIV-1_IIIb_ (5 ng/ml of HIV-1 gag p24) with increasing concentrations of M48U1 formulated in 0.25% HEC for one hour at 37 °C in RPMI 1640 medium. This mixture was added to PBMCs activated with IL-2 and PHA for a two-hour incubation at 37 °C. To evaluate the antiviral effect of M48U1, the level of HIV-1 gag p24 protein in the culture supernatant was determined at seven days post-infection using an ELISA. A significant decrease (p < 0.05; Mann Whitney test) in the HIV-1 p24 level was found (Fig. 1). The IC_50_ of M48U1 was calculated to be 43.4 nM (95% CI 17.8–106.72) and 33.2 nM (95% CI 22.65–48.64) in cultures infected with HIV-1_BaL_ (Fig. 1A) and HIV-1_IIIb_ (Fig. 1B), respectively.

Although the antiretroviral activity of tenofovir has been well-characterized^28^,^33^,^51^,^52^, we evaluated the effects of tenofovir formulated in 0.25% HEC specifically on HIV-1 replication under the same experimental conditions as above. We used increasing concentrations of tenofovir formulated in 0.25% HEC to treat activated PBMCs challenged with HIV-1_BaL_ or HIV-1_IIIb_. For both strains assessed, the level of HIV-1 p24 in the cell culture supernatant on day 7 post-infection showed a concentration-dependent inhibition of infection by tenofovir. The IC_50_ of tenofovir was found to be 1.22 μM (95% CI 1.1–1.4) and 1.08 μM (95% CI 0.68–1.7) for HIV-1_BaL_ (Fig. 1C) and HIV-1_IIIb_ (Fig. 1D), respectively.

To rule out the possibility that the antiviral effects were related to the formulation of the drugs in 0.25% HEC, we analyzed whether M48U1 in 0.25% HEC or tenofovir in 0.25% HEC may induce adverse effects on activated PBMCs. Increasing concentrations of M48U1 or tenofovir in 0.25% HEC were added to cell cultures, and the viability was determined by the Trypan Blue exclusion assay. The viability of the activated PBMCs was not affected by either of the drugs formulated in 0.25% HEC compared to untreated PBMC cell cultures, showing a CC_50_ value > 10 μM for M48U1 and a CC_50_ value > 20 μM for tenofovir (Supplementary Figure 1).

### Individual infections with HIV-1 reference strains, viral isolates from patients and transmitted/founder infectious molecular clones were effectively inhibited by treatment with M48U1 or tenofovir in 0.25% HEC

**Figure 2:**
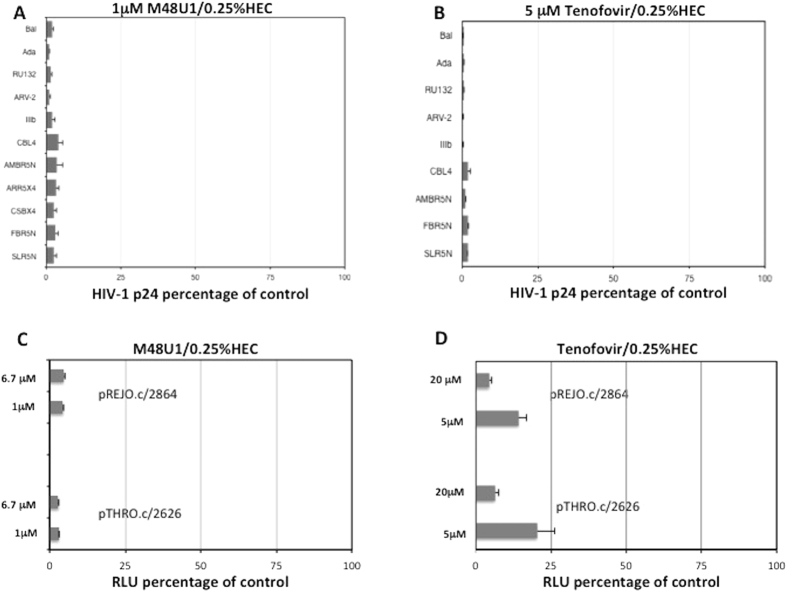
M48U1 in 0.25% HEC and tenofovir in 0.25% HEC each inhibited several laboratory strains and patient-derived HIV-1 isolates. Treatment with either 1 μM M48U1/0.25% (**A**) or 5 μM tenofovir/0.25% HEC (**B**) inhibited the replication of all tested laboratory and patient-derived strains. The HIV-1 strain tropism was determined with a sequencing analysis for the patient-derived strains. The combination of the two HIV-1 drugs inhibited HIV-1 replication independent of the HIV-1 strain tropism. HIV-1 replication was monitored by the HIV-1 p24 ELISA assay using cell culture supernatants obtained on day 7 post-infection. The data were expressed as the means ± standard deviations (±SD) of the level of HIV-1 gag p24 relative to untreated controls (set to 100%) for every HIV-1 strain. Three experiments in triplicate were performed. Analysis of antiretroviral activity of 1 μM M48U1 in 0.25% HEC (**C**) or 5 μM tenofovir in 0.25% HEC (**D**) performed in TZM-bl cells infected with pREJO.c/2864 and pTHRO.c/2626 T/F IMCs. The percentage of infection of each T/F was calculated comparing the relative luminescence units (RLUs) of the virus control wells, after subtraction of cell control RLUs and the data were expressed as the means ± standard deviations (±SD). Each sample was performed in triplicate and was repeated in three independent experiments.

Because different HIV-1 strains may have different IC_50_ values for M48U1 and tenofovir, we chose a concentration of each drug that inhibited over 90% of HIV-1_BaL_ and HIV-1_IIIb_ replication. In the next series of experiments, we tested the antiretroviral activity of 1 μM M48U1 in 0.25% HEC or 5 μM tenofovir in 0.25% HEC in PBMCs challenged with different HIV-1 reference and patient strains. In addition to R5-tropic HIV-1_BaL_ and X4-tropic HIV-1_IIIb,_ four classical laboratory strains, including R5-tropic HIV-1_ADA_ and HIV-1_RU132,_ R5/X4 dual-tropic HIV-1_ARV-2_ and X4-tropic HIV-1_CBL4,_ were used to infect activated PBMCs. By analyzing the level of p24 protein in the cell supernatant (Fig. 2A,B) we found that each drug suppressed the replication of all HIV-1 strains tested (p < 0.05).

We also isolated and characterized HIV-1 subtype B and C strains from five patients: three naïve patients and two patients with therapeutic failure and resistance to certain protease and reverse transcriptase inhibitors. Sequence analysis demonstrated that the naïve patients were infected with R5-tropic HIV-1 subtype B (HIV-1_AMBR5N_) and subtype C strains (HIV-1_FBR5N_ and HIV-1_SLR5N_), whereas the two patients with cART failure were infected with R5/X4-tropic HIV-1_ARR5X4_ or X4-tropic HIV-1_CSBX4_ B subtype strains. Treatment with 1 μM M48U1 in 0.25% HEC (Fig. 2A) prevented infection of PBMCs with all HIV-1 subtype B and C isolates (p < 0.05; Mann Whitney test). On the other hand, 5 μM tenofovir in 0.25% HEC (Fig. 2B) was tested only with the HIV-1 subtype B and C strains that were isolated from three naïve patients, in the absence of mutations (inducing resistance to NRTI). Tenofovir in 0.25% HEC inhibited viral replication in all three cases (p < 0.05; Mann Whitney test), demonstrating that this formulation prevented HIV-1 infection of activated PBMCs with both laboratory and patient-derived strains.

Because the use of transmitted/founder infectious molecular clones (T/F IMCs), would be both informative for understanding HIV-1 transmission and provide new opportunities to find drugs that block the earliest stages of HIV-1 transmission, we also analysed the antiretroviral activity of 1 or 6.7 μM M48U1 in 0.25% HEC and 5 or 20 μM tenofovir in 0.25% HEC in TZM-bl cells challenged separately with two T/F IMCs, pREJO.c/2864 and pTHRO.c/2626. As shown in Fig. 2C,D, a clear decrease in viral infectivity was observed when experiments were performed in the presence of M48U1 or tenofovir). A dose-dependent response activity was found for tenofovir (Fig. 2D) but not for M48U1 (Fig. 2C), which showed the maximum level of inhibition at both concentrations of 1 and 6.7 μM. In fact, treatment with 1 or 6.7 μM of M48U1 in 0.25% HEC induced a reduction a 96.7% (±0.07) and 96.6% (±0.05) reduction in viral replication, respectively for pREJO.c/2864 or 95% (±0.09 or ±0.15) for pTHRO.c/2626. A reduction in viral replication of pREJO.c/2864 of 86.8% (±3.08) and 95% (±1.28) was observed in the presence of 5 or 20 μM tenofovir in 0.25% HEC respectively. The same concentration of tenofovir decreased viral replication of pTHRO.c/2626 by 78% (±0.51) and 93% (±2.04), which supports our data obtained with strains belonging to HIV-1 subtypes B and C.

### Synergistic effects of M48U1 and tenofovir formulated in 0.25% HEC on HIV-1 replication

**Figure 3:**
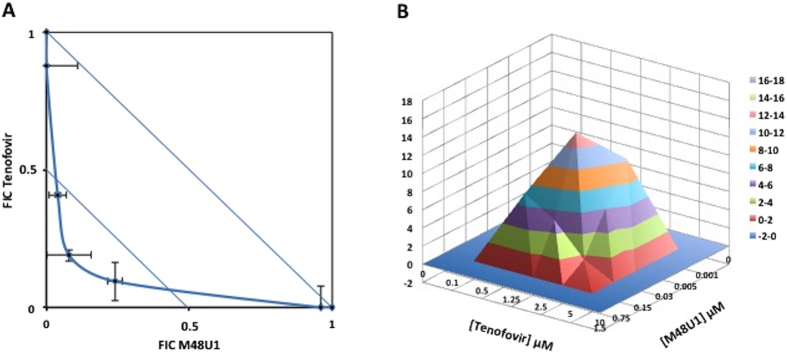
The combined formulation of M48U1 and tenofovir in 0.25% HEC exhibited synergistic antiretroviral activity against infection with R5-tropic HIV-1_BaL_. Combinations of M48U1/tenofovir (0 μM/10 μM; 0.001 μM/5 μM; 0.005 μM/2.5 μM; 0.03 μM/1.25 μM; 0.15 μM/0.5 μM; 0.75 μM/0.1 μM and 1.5 μM/0 μM were tested in PBMCs challenged with the HIV-1_BaL_ strain and isobologram determination was performed (**A**). In particular, the M48U1/tenofovir combinations 0.005 μM/2.5 μM; 0.03 μM/1.25 μM; 0.15 μM/0.5 μM were synergistic. Data for each compound are reported as the fractional inhibitory concentration (FIC), calculated by dividing the IC_50_ of compound “X” in combination with compound “Y” by the IC_50_ of compound “X” alone. The solid and broken lines represent the unit lines for an FIC equal to 1 and 0.5, respectively. The FIC index, corresponding to the sum of the FIC values for compounds “X” and “Y,” is indicated for every combination of concentrations. An FIC index between 0.5 and 0.9 is suggestive of partial synergism, whereas an FIC index < 0.5 indicates significant synergism. In panel **B**, MacSynergy II analysis is shown. A 3D synergy plot, obtained at 95% confidence interval represents the synergism between M48U1 and tenofovir at different concentrations. Peaks above the theoretical additive plane (level 0) indicate synergy, whereas peaks below the plane indicate antagonism. Statistically significant synergies are shown using different colors, from red to pink, for each range of Synergy volume. Pink color indicates the strongest synergy. The synergy value volume was determined at 68.44 μM^2^% indicating the presence of synergy^48^. The data should be interpreted as follows: values between 25 and 50 μM^2^% minor but significant synergy, between 50 and 100 μM^2^% synergy, >100 μM^2^% strong synergy. These results were obtained from three independent experiments in triplicate.

**Figure 4:**
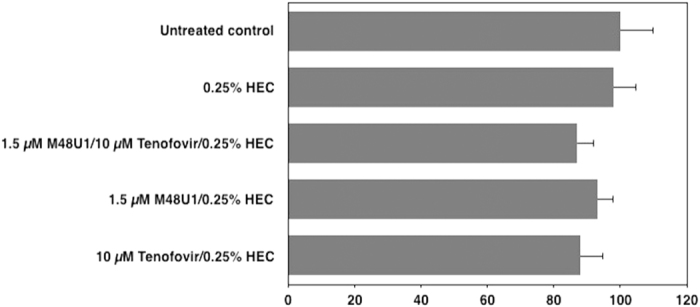
The combination of M48U1 plus tenofovir was not toxic to PBMCs. Activated PBMCs were treated with the higher concentrations of M48U1 and/or tenofovir tested in the earlier studies. HIV-1 replication was monitored by Trypan blue technique on day 7 post-infection. The data were expressed as the means ± standard deviations (±SD) of viable cells relative to untreated controls (set to 100%) obtained from three independent experiments performed in triplicate.

Previous studies demonstrated that tenofovir acts synergistically with several antiretroviral drugs, including some NRTIs, PI and entry inhibitors^28^,^33^. We therefore explored whether treatment with M48U1 and tenofovir combined in 0.25% HEC has a synergistic effect on viral replication in an *in vitro* cellular model of activated PBMCs infected with the R5-tropic HIV-1_BaL_ strain. To do so, the combined activity of tenofovir and M48U1 in serial concentrations was evaluated by examining the amount of HIV-1 p24 present in the cell supernatant on day 7 and synergy was determined with the isobologram. Figure 3A shows the isobologram, where the FIC values achieved for several combinations of concentrations of the two antiretroviral drugs demonstrated their synergistic activity. The FIC index was <0.5, clearly indicating the presence of synergistic effects between the drugs. A parallel analysis with the MacSynergy II^48^ program demonstrated synergism between M48U1 and tenofovir, reaching a peak of 14.52 μM^2^ synergy volume with a total 68.44 μM^2^% of synergy volume thus confirming the synergism between the two drugs (Fig. 3B). The Trypan Blue exclusion assay was employed to determine whether these co-treatments could exert cytotoxic effects that would explain the antiretroviral effects of the drugs. None of combinations with the higher concentrations of these antiretroviral compounds exhibited significant effects on the viability of activated PBMCs (p > 0.05; one-way ANOVA test; Fig. 4), indicating that the synergistic antiviral effect was not associated with cytotoxicity but was likely related to the inhibitory effects of M48U1 and tenofovir on two distinct phases of the HIV-1 replication cycle.

### M48U1 and tenofovir inhibited the HIV-1 replication in cervicovaginal histocultures

In the previous experiments, we demonstrated that tenofovir and M48U1 exerted synergistic antiretroviral effects when formulated in HEC using an activated PBMC model. We next investigated the antiretroviral effects of M48U1 and/or tenofovir alone or in combination in a 0.25% HEC vehicle gel using an organotypic model of cervicovaginal epithelial tissue to determine the effectiveness of these formulations as a vaginal mucosal microbicide. Different formulations of M48U1 (0.67 μM or 6.7 μM M48U1), tenofovir (2 μM or 20 μM) or the combination (0.67 μM M48U1 plus 2 μM tenofovir or 6.7 μM M48U1 plus 20 μM tenofovir) were applied in a 0.25% HEC hydrogel formulation to the apical surface of cervicovaginal cell cultures that were subsequently challenged with HIV-1_BaL_ (25 ng HIV-1 p24/tissue) for 24 hours. We have tested these higher concentrations of M48U1 (6.7 μM) and tenofovir (20 μM) because the cervicovaginal tissues and PBMCs have different susceptibilities to HIV-1 infection and treatment. Both HIV-1 and antiretroviral concentrations were increased according to several papers^31^,^53^,^54^. Moreover, this experimental analysis focused on the R5-tropic HIV-1 strain because the transmission of HIV-1 by sexual intercourse is almost exclusively determined by the CCR5 tropism^10^,^39^.

**Figure 5:**
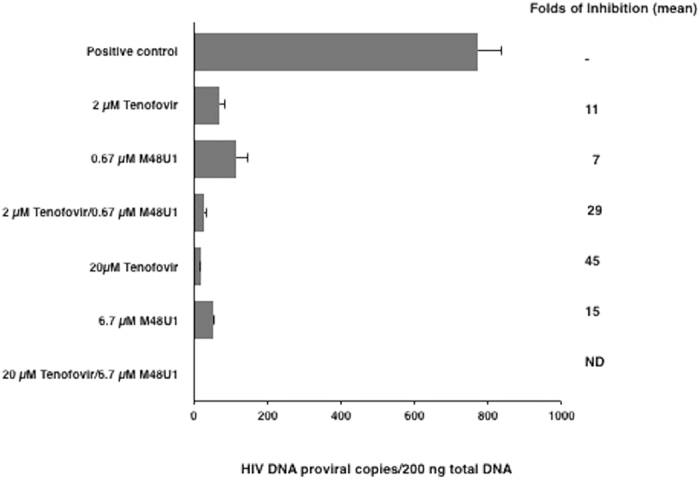
M48U1 plus tenofovir treatment of human cervicovaginal histocultures effectively prevented the replication of HIV-1_BaL_. Full thickness cervicovaginal histocultures were treated with two different concentrations of M48U1 (0.67 μM or 6.7 μM) with or without tenofovir at 2 μM or 20 μM formulated in 0.25% HEC hydrogel. In the presence of these drugs, the tissue samples were challenged with the HIV-1_BaL_ strain (25 ng p24/well) for 24 hours. Total DNA was purified from the tissues on day 4 post-infection and was analysed by qPCR. When 6.7 μM M48U1 was used with 20 μM tenofovir, no proviral DNA was detectable. The data were expressed as the means ± standard deviations (±SD) of the number of copies of proviral DNA/200 ng total DNA and the fold-inhibition was calculated as the mean with respect to HIV-1-infected samples treated with HEC hydrogel (0.25%) without any antiretroviral drug. Three experiments in triplicate or duplicate were performed. ND: not detected.

The presence of HIV-1 infection was evaluated with SYBR green-based quantitative real time PCR technique to determine the content of HIV-1 proviral DNA on day 4 post-infection using LTR-specific primers. The samples treated with 6.7 μM M48U1 or 20 μM tenofovir showed a decrease in the amount of proviral HIV-1 DNA by approximately 15-fold and 45-fold, respectively. This HIV-1 replication inhibition was detectable to a lesser extent when 0.67 μM M48U1 and 2 μM tenofovir concentrations were employed (7- and 11-fold, respectively). The combination of M48U1 and tenofovir elicited a further decrease in HIV-1 DNA compared to either alone. In fact, when the combination of the higher concentrations was used (6.7 μM M48U1 plus 20 μM tenofovir), no HIV-1 proviral DNA was detected by the quantitative PCR assay, and the combination of 0.67 μM M48U1 plus 2 μM tenofovir in 0.25% HEC induced a 29-fold decrease in the HIV-1 proviral DNA burden (Fig. 5).

Because the EpiVaginal cell model is a cellular system that is also suitable for predicting the vaginal toxicity of microbicides, we also assessed the irritation potential of M48U1 and/or tenofovir treatment. We treated the cervicovaginal tissues with 6.7 μM M48U1, 20 μM tenofovir, or the 6.7 μM M48U1 plus 20 μM tenofovir combination, all of which were formulated in 0.25% HEC hydrogel, for 1, 4, 18 and 96 hours at 37 °C. The samples were analysed for the cellular metabolic activity of living cells by the MTT kit and for the accumulation of dead cells by evaluating the LDH release. In addition, we evaluated the release of IL-1α to determine the inflammatory cell activation. Table 1 showed that the M48U1 and/or tenofovir are not toxic at tested concentrations (p > 0.05; one-way ANOVA test) as well as LDH and IL-1α proinflammatory cytokine release was not significantly increased by the drug treatments (p > 0.05; two-way ANOVA test followed by Bonferroni test) with respect to untreated controls.

## Discussion

This report investigated the antiretroviral activity of HEC-formulated tenofovir and M48U1 in both *in vitro* and *ex vivo* cellular models, including activated PBMCs, TZM-bl cells and a human organotypic model of cervicovaginal tissue. We observed that: (i) M48U1 and tenofovir suppressed infection of activated PBMCs with several HIV-1 reference strains and viral isolates from patients; (ii) M48U1 and tenofovir inhibited infection of TZM-bl cells with T/F IMCs; (iii) M48U1 and tenofovir exhibited synergistic antiretroviral effects; and (iv) M48U1 and/or tenofovir treatments inhibited HIV-1 R5 strain replication in an *ex vivo* cervicovaginal tissue model without any significant effect on tissue viability.

M48U1 formulated in HEC effectively and reliably inhibited infection of PBMCs with laboratory HIV-1 strains and with HIV-1 subtype B and C strains isolated from naïve and cART-treated patients. To develop a microbicide able to prevent HIV-1 transmission, it is essential to evaluate the activity of M48U1 on several primary virus strains, including transmitted/founder strains. Certain commonly used, laboratory strains are highly sensitive to inhibition of infection and thus do not adequately reflect the broad spectrum of inhibition observed for primary viruses of various clades. Suppressing the replication of HIV-1 subtype C viruses is highly relevant because these viruses account for approximately 50% of all infections worldwide and they are the most prevalent in sub-Saharan Africa^55^. In addition, M48U1 also inhibited T/F IMCs, which were derived from patients with acute infection and thus represent transmitted viruses acquired through a mucosal site *in vivo*. The broad antiretroviral activity of M48U1 and the synergistic activity of the M48U1 combined with tenofovir in the prevention of HIV infection could overcome the variability of HIV strains over time, as virus isolates from later stages of infection can differ substantially from the earlier virus population and in particular from the respective transmitted virus strain(s). Overall, our findings demonstrated that the inhibition of HIV replication was not dependent on the subtype, HIV-1 tropism or stage of infection. Our analysis of the antiretroviral activity of tenofovir and M48U1 showed that tenofovir has lower antiviral effects on IMCs with respect to laboratory and wild type HIV-1 strains. We have already shown that IMCs generally showed higher IC_50_ values in neutralization assays^44^ thus suggesting a possible higher resistance to some neutralizing compounds. Differences in neutralization sensitivity could also be due to technical manipulation of the different viral strains as IMCs have been produced in the context of a proviral background. Other studies have reported that different ratios of backbone and env-plasmids transfected in host cells were found to give rise to viral particles endowed with different envelope features, such as the proportion of env protein cleavage and the level of gp120 surface expression; such changes in envelope features were found to affect viral infectivity, and, possibly, antibody reactivity^56^. In addition, the host cells are known to impact biochemical and structural features of virus particles, e.g. in terms of protein processing, folding and glycosylation patterns^57^. Indeed, in our study, diversity in the sensitivity to neutralization could be ascribed to the effect of the different host cells (293 T cells vs PBMC) as well.

M48U1 is a CD4 mimetic peptide (27 mer) that binds gp120 with higher affinity than that of the native CD4 protein^34^,^35^. This property is due to the presence of a protrusion at amino acid 23 of M48U1, which stabilizes the Phe43 cavity, a critical element in the interaction between gp120 and CD4^58^. The antiretroviral activity of M48U1 was originally attributed to its inhibition of viral entry. However, another investigation indicated that this peptide not only acts in the first phase of HIV-1 entry but also elicits gp120 shedding through binding to the gp120 exposed on the cell membrane to induce the release of functionally defective HIV-1 particles^59^. This peptide represents a valuable new tool with respect to soluble CD4 (sCD4), which was only able to inhibit specific HIV-1 strains and, under some experimental conditions, actually enhanced infection, especially with HIV-1 wild-type strains^60^,^61^,^62^. Intriguingly, M48U1 can suppress both cell-free and cell-to-cell HIV-1 infection^61^, suggesting that this drug may be a good candidate as a vaginal microbicide, especially because heterosexual transmission through the vaginal mucosa is mainly due to cell-free viruses but can also involve cell-to-cell HIV-1 transmission related to the transfer of HIV-1-infected lymphocytes and macrophages, detectable in the semen, to the cells present in the vaginal submucosa^63^,^64^. As M48U1 is stable in vaginal fluids and is very soluble at acidic pH^30^,^36^, its possible use as a single microbicide was proposed by a study in a simian model using an M48U1 vaginal gel, where M48U1 prevented SHIV-1_162P3_ infection in Cynomolgus macaques^30^. In parallel, we analysed the effects of tenofovir formulated in HEC on activated PBMCs and in TZM-bl cells. Tenofovir is an inhibitor of reverse transcriptase, which acts as a DNA chain terminator. Its modified form, tenofovir disoproxil fumarate, is currently used in cART regimens^65^,^66^. As expected^54^,^67^, tenofovir formulated in HEC maintained its antiretroviral activity in both cell models.

We evaluated tenofovir as an antiretroviral drug for use with M48U1. Treatment with M48U1 plus tenofovir showed a clear synergism in the inhibition of HIV-1 replication in activated PBMCs, probably due to the different targets of these molecules in the HIV-1 replication cycle. Note that tenofovir has exhibited synergistic antiretroviral activity with many other antiretroviral molecules^28^,^33^,^51^,^52^, including all non-nucleoside reverse transcriptase inhibitors (NNRTIs), some protease inhibitors (amprenavir, nelfinavir), the entry inhibitor Griffithsin and CADA. Combination treatment with two or more antiretroviral molecules is the current strategy for increasing the antiretroviral activity of microbicides. Interestingly, the advent of microbicide treatment has occurred in tandem with the progressive improvement of systemic antiretroviral therapy, in which, the combinations of different antiretroviral drugs replaced the single antiretroviral compound treatment. In fact, microbicide treatment with a single drug has various weaknesses, including the failure of HIV-1 inhibition in the presence of specific resistant HIV-1 strains and the limited effectiveness of single treatment, which may not be potent enough to inhibit HIV-1 transmission. In contrast, pharmacological approaches using multiple antiretroviral drugs have several advantages, including the possibility of interfering with different phases of the viral replication cycle, the ability to use lower concentrations of individual drugs, which can decrease the risk of physical and functional damage to the vaginal epithelia, and the potential for interfering with the replication of drug-resistant strains.

In the last series of experiments in the present study, M48U1 plus tenofovir formulated in HEC effectively suppressed HIV-1 replication in *ex vivo* human cervicovaginal histocultures. Quantitative analysis of the proviral DNA indicated that the combination of 0.67 μM M48U1 plus 2 μM tenofovir exhibited a high level of inhibition of HIV-1 replication, which was relatively similar to the effects of single drug treatment at a 10-fold higher concentration. In addition, our results indicate that the combination of M48U1 and tenofovir was not toxic to the human cervicovaginal histocultures. This finding is in line with the absence of M48U1 toxicity and the stability of the peptide at vaginal pH in a simian model^30^ as well as with the extensive studies on tenofovir both *in vivo* and *ex vivo*, which showed that this drug has consistent tolerability and safety in different gel formulations^26^,^54^. Tenofovir was the first reverse transcriptase inhibitor to be successfully used as a microbicide in topical gel formulations in human trials^24^, and an analysis of tenofovir treatment using a vaginal gel demonstrated the presence of tenofovir at high concentrations in vaginal tissues and in cellular models^68^,^69^. Importantly, tenofovir can impair HIV-1 transmission through an additional mechanism inhibiting HSV-2 infection^24^,^68^,^70^. In the CAPRISA 004 trial, HSV-2 infections decreased by 51% in patients treated with tenofovir^24^,^68^,^70^. This anti-HSV-2 effect is important to counteract the spread of HIV-1, because HSV-2 infection induces genital epithelial ulcers that increase the risk of HIV-1 transmission due to the disruption of mucosal barriers and induction of inflammation^64^,^71^. Epidemiological observations demonstrated that the risk for HIV-1 infection transmission was twice as high for women infected with HSV-2^72^. In the present study, M48U1 and/or tenofovir were combined in HEC hydrogel, which is considered to be a universal vehicle gel, with no toxicity evident in cell cultures^54^,^67^. Our data confirmed the absence of cell damage or cell death induced by 0.25% HEC in both PBMCs and in organotypic cervicovaginal tissues. In addition, as expected^54^, the 0.25% HEC formulation did not inhibit the diffusion of tenofovir and M48U1 to allow them to interact with cells and HIV-1. The combined use of tenofovir and M48U1 is valuable as a potential new microbicide treatment, with several advantages including drug synergism, the absence of toxicity and the possibility to interfere in two different stages of HIV-1 replication. In particular, the level of synergy could allow for the use of a lower amount of each drug to give an optimal antiretroviral effect with a possible reduction of drug side effects. In conclusion, these results suggest that M48U1 plus tenofovir treatment may be considered as a useful topical microbicide to counteract heterosexual transmission of HIV-1.

## Additional Information

**How to cite this article**: Musumeci, G. *et al*. M48U1 and Tenofovir combination synergistically inhibits HIV infection in activated PBMCs and human cervicovaginal histocultures. *Sci. Rep.* **7**, 41018; doi: 10.1038/srep41018 (2017).

**Publisher's note:** Springer Nature remains neutral with regard to jurisdictional claims in published maps and institutional affiliations.

## Supplementary Material

Supplementary Figure 1

## Figures and Tables

**Table 1:** Evaluation of the viability (MTT assay), LDH release and IL-1α release in the EpiVaginal tissue model treated with M48U1 and/or tenofovir.

	Viability (%)	LDH release (Abs)	IL-1α Release (pg/mL)
Untreated (1 hour)	100 + 5	0.211 ± 0.02	7.9 ± 0.9
20 μM tenofovir/0.25% HEC (1 hour)	95 + 4	0.207 ± 0.06	7.8 ± 0.8
6.7 μM M48U1/0.25% HEC (1 hour)	98 + 6	0.222 ± 0.05	8.0 ± 1.0
20 μM tenofovir + 6.7 μM M48U1/0.25% HEC (1 hour)	97 + 5	0.218 ± 0.05	8.4 ± 0.7
Untreated (4 hours)	100 + 4	0.368 ± 0.05	9.7 ± 1.3
20 μM tenofovir/0.25% HEC (4 hours)	96 + 6	0.381 ± 0.05	9.9 ± 1.8
6.7 μM M48U1/0.25% HEC (4 hours)	98 + 7	0.364 ± 0.01	10.5 ± 1.1
20 μM tenofovir + 6.7 μM M48U1/0.25% HEC (4 hours)	97 + 5	0.337 ± 0.01	10.3 ± 1.6
Untreated (18 hours)	100 + 8	1.179 ± 0.13	34.1 ± 2.6
20 μM tenofovir/0.25% HEC (18 hours)	88 + 4	0.922 ± 0.2	36.5 ± 1.2
6.7 μM M48U1/0.25% HEC (18 hours)	89 + 5	0.996 ± 0.2	33.8 ± 5.4
20 μM tenofovir + 6.7 μM M48U1/0.25% HEC (18 hours)	91 + 6	1.103 ± 0.1	34.6 ± 4.2
Untreated (96 hours)	100 + 6	1.557 ± 0.08	61.9 ± 5.8
20 μM tenofovir/0.25% HEC (96 hours)	104 + 9	1.498 ± 0.13	59.7 ± 7.7
6.7 μM M48U1/0.25% HEC (96 hours)	91 + 7	1.544 ± 0.15	62.2 ± 9.3
20 μM tenofovir + 6.7 μM M48U1/0.25% HEC (96 hours)	98 + 9	1.631 ± 0.21	64.0 ± 8.7

## References

[b1] UNAIDS/WHO 2016. Report on global AIDS epidemic. Geneva UNAIDS (2016).

[b2] Shattock R. J. & Rosenberg Z. Microbicides: topical prevention against HIV-1. Cold Spring Harb. Perspect. Med. 2, a007385 (2012). 22355798 10.1101/cshperspect.a007385 PMC3281595

[b3] UNAIDS/WHO 2013. Fact sheet n°334. Geneva UNAIDS (2013).

[b4] Cohen M. S. HIV-1 and sexually transmitted diseases: lethal synergy. Top. HIV-1 Med. 12, 104–107 (2004). 15516707

[b5] Higgins J. A., Hoffman S. & Dworkin S. L. Rethinking gender, heterosexual men, and women’s vulnerability to HIV-1/AIDS. Am. J. Public Health. 100, 435–445 (2010). 20075321 10.2105/AJPH.2009.159723 PMC2820057

[b6] Lohse N. . Survival of persons with and without HIV-1 infection in Denmark, 1995-2005. Ann. Intern. Med. 146, 87–95 (2007). 17227932 10.7326/0003-4819-146-2-200701160-00003

[b7] Marsden M. D. & Zack J. A. Eradication of HIV-1: current challenges and new directions. J. Antimicrob. Chemother. 63, 7–10 (2009). 18984648 10.1093/jac/dkn455 PMC2721701

[b8] Cohen M. S. . HPTN 052 Study Team. Prevention of HIV-1 infection with early antiretroviral therapy. N Engl J Med. 365, 493–505 (2011). 21767103 10.1056/NEJMoa1105243 PMC3200068

[b9] Cohen Y. Z. & Dolin R. Novel HIV-1 vaccine strategies: overview and perspective. Ther. Adv. Vaccines. 1, 99–112 (2013). 24757518 10.1177/2051013613494535 PMC3967667

[b10] Shattock R. J. & Moore J. P. Inhibiting sexual transmission of HIV-1 infection. Nat. Rev. Microbiol. 1, 25–34 (2003). 15040177 10.1038/nrmicro729

[b11] Lederman M. M., Offord R. E. & Hartley O. Microbicides and other topical strategies to prevent vaginal transmission of HIV-1. Nat. Rev. Immunol. 6, 371–382 (2006). 16639430 10.1038/nri1848

[b12] Morris G. C. & Lacey C. J. Microbicides and HIV-1 prevention: lessons from the past, looking to the future. Curr. Opin. Infect. Dis. 23, 57–63 (2010). 19918175 10.1097/QCO.0b013e328334de6d

[b13] Foss A. M., Vickerman P. T., Alary M. & Watts C. H. How much could a microbicide’s sexually transmitted infection efficacy contribute to reducing HIV-1 risk and the level of condom use needed to lower risk? Model estimates. Sex Transm. Infect. 85, 276–282 (2009). 19208696 10.1136/sti.2008.032458

[b14] Smith R. J., Bodine E. N., Wilson D. P. & Blower S. M. Evaluating the potential impact of vaginal microbicides to reduce the risk of acquiring HIV-1 in female sex workers. AIDS 19, 413–421 (2005). 15750395 10.1097/01.aids.0000161771.44276.92

[b15] Chen B. A. . Phase 1 Safety, Pharmacokinetics, and Pharmacodynamics of Dapivirine and Maraviroc Vaginal Rings: A Double-Blind Randomized Trial. J Acquir Immune Defic Syndr. 70, 242–249 (2015). 26034880 10.1097/QAI.0000000000000702 PMC4607587

[b16] Cohen M. S. . Prevention of HIV-1 infection with early antiretroviral therapy. N. Engl. J. Med. 365, 493–505 (2011). 21767103 10.1056/NEJMoa1105243 PMC3200068

[b17] Baeten J. M., Haberer J. E., Liu A. Y. & Sista N. Preexposure prophylaxis for HIV-1 prevention: where have we been and where are we going? J. Acquir. Immune Defic. Syndr. 63, S1229–1230 (2013). 10.1097/QAI.0b013e3182986f69 PMC3710117 23764623

[b18] Miller V. & Grant M. R. Regulatory considerations for antiretroviral prophylaxis to prevent HIV-1 acquisition. Clin. Pharmacol. Therap. 96, 153–155 (2014). 25056397 10.1038/clpt.2014.114

[b19] Maszynski P. Halt to microbicide trial sets back AIDS research. BMJ 334, 276 (2007). 10.1136/bmj.39118.473356.DB PMC1796710 17297700

[b20] Skoler-Karpoff S. . Efficacy of Carraguard for prevention of HIV-1 infection in women in South Africa: a randomised, double-blind, placebo controlled trial. Lancet 372, 1977–1987 (2008). 19059048 10.1016/S0140-6736(08)61842-5

[b21] Van Damme L., Corneli A. & Ahmed K. FEM-PreP Study Group. Pre-exposure prophylaxis for HIV-1 infection among african women. N. Engl. J. Med. 367, 411–422 (2012). 22784040 10.1056/NEJMoa1202614 PMC3687217

[b22] Singh O., Garg T., Rath G. & Goyal A. K. Microbicides for the treatment of sexually transmitted diseases. J. Pharm. 2014, 352425 (2014). 10.1155/2014/352425 PMC4590794 26556193

[b23] Abdool Karim S. S. Results of effectiveness trials of PRO 2000 gel: lessons for future microbicide trials. Future Microbiol. 5, 527–529 (2010). 20353292 10.2217/fmb.10.29

[b24] Abdool Karim Q. . CAPRISA 004 Trial Group. Effectiveness and safety of tenofovir gel, an antiretroviral microbicide, for the prevention of HIV-1 infection in women. Science 329, 1168–1174 (2010). 20643915 10.1126/science.1193748 PMC3001187

[b25] Grant R. M. . Preexposure chemoprophylaxis for HIV-1 prevention in men who have sex with men. N. Engl. J. Med. 363, 2587–2599 (2010). 21091279 10.1056/NEJMoa1011205 PMC3079639

[b26] Marrazzo J. M. . Tenofovir-based preexposure prophylaxis for HIV-1 infection among African women. N. Engl. J. Med. 372, 509–518 (2015). 25651245 10.1056/NEJMoa1402269 PMC4341965

[b27] Lin P. F. . A small molecule HIV-1 inhibitor that targets the HIV-1 envelope and inhibits CD4 receptor binding. Proc. Natl. Acad. Sci. USA. 100, 11013–11018 (2003). 12930892 10.1073/pnas.1832214100 PMC196918

[b28] Vermeire K. . CADA, a novel CD4-targeted HIV-1 inhibitor, is synergistic with various anti-HIV-1 drugs *in vitro*. AIDS 18, 2115–2125 (2004). 15577644 10.1097/00002030-200411050-00003

[b29] Martin G. . A simple one-step method for the preparation of HIV-1 envelope glycoprotein immunogens based on a CD4 mimic peptide. Virology 381, 241–250 (2008). 18835005 10.1016/j.virol.2008.08.039 PMC2645002

[b30] Dereuddre-Bosquet N. . MiniCD4 microbicide prevents HIV-1 infection of human mucosal explants and vaginal transmission of SHIV-1 (162P3) in cynomolgus macaques. PLoS Pathog. 8, e1003071 (2012). 23236282 10.1371/journal.ppat.1003071 PMC3516572

[b31] Bon I. . Peptide-derivatized SB105-A10 dendrimer inhibits the infectivity of R5 and X4 HIV-1 strains in primary PBMCs and cervicovaginal histocultures. PLoS One. 8, e76482 (2013). 24116111 10.1371/journal.pone.0076482 PMC3792046

[b32] Klasse. P. J., Shattock R. & Moore J. P. Antiretroviral drug-based microbicides to prevent HIV-1 sexual transmission. Annu Rev Med. 59,455–471 (2008). 17892435 10.1146/annurev.med.59.061206.112737

[b33] Ferir G., Palmer K. E. & Schols D. Synergistic activity profile of griffithsin in combination with tenofovir, maraviroc and enfuvirtide against HIV-1 clade C. Virology. 417, 253–258 (2011). 21802104 10.1016/j.virol.2011.07.004

[b34] Van Herrewege Y. . CD4 mimetic miniproteins: potent anti-HIV-1 compounds with promising activity as microbicides. J. Antimicrob. Chemoth. 61, 818–826 (2008). 10.1093/jac/dkn042 18270220

[b35] Acharya P. . Structural basis for highly effective HIV-1 neutralization by CD4-mimetic miniproteins revealed by 1.5 Å cocrystal structure of gp120 and M48U1. Structure. 21, 1018–1029 (2013). 23707685 10.1016/j.str.2013.04.015 PMC4140785

[b36] Bouchemal K. . Thermosensitive and mucoadhesive pluronic-hydroxypropylmethylcellulose hydrogel containing the mini-CD4 M48U1 is a promising efficient barrier against HIV-1 diffusion through macaque cervicovaginal mucus. Antimicrob. Agents Chemother. 59, 2215–2222 (2015). 25645853 10.1128/AAC.03503-14 PMC4356805

[b37] Svicher V. . Performance of genotypic tropism testing in clinical practice using the enhanced sensitivity version of Trofile as reference assay, results from the OSCAR Study Group. New Microbiol. 33, 195–206 (2010). 20954437

[b38] Gartner S. & Popovic M. Virus Isolation and production in HIV-1 techniques (Eds Aldovini A. & Walker B. D.) pp 53–69 (Stockton Press, 1990).

[b39] Salazar-Gonzalez J. F. . Genetic identity, biological phenotype, and evolutionary pathways of transmitted/ founder viruses in acute and early HIV-1 infection. J. Exp. Med. 206, 1273–1289 (2009). 19487424 10.1084/jem.20090378 PMC2715054

[b40] Keele B. F. . Identification and characterization of transmitted and early founder virus envelopes in primary HIV-1 infection. Proc. Natl. Acad. Sci. USA 105, 7552–7557 (2008). 18490657 10.1073/pnas.0802203105 PMC2387184

[b41] Ochsenbauer C. . Generation of transmitted/founder HIV-1 infectious molecular clones and characterization of their replication capacity in CD4 T lymphocytes and monocyte-derived macrophages. J. Virol. 86, 2715–2728 (2012). 22190722 10.1128/JVI.06157-11 PMC3302286

[b42] Martin G. . Stabilization of HIV-1 envelope in the CD4-bound conformation through specific cross-linking of a CD4 mimetic. J. Biol. Chem. 286, 21706–21716 (2011). 21487012 10.1074/jbc.M111.232272 PMC3122227

[b43] Bouchemal K. . Note on the formulation of thermosensitive and mucoadhesive vaginal hydrogels containing the miniCD4 M48U1 as anti-HIV-1 microbicide. Int. J. Pharm. 454, 649–652 (2013). 23500765 10.1016/j.ijpharm.2013.02.055

[b44] Miglietta R. . Synergy in monoclonal antibody neutralization of HIV-1 pseudoviruses and infectious molecular clones. J. Transl. Med. 12, 346 (2014). 25496375 10.1186/s12967-014-0346-3 PMC4274758

[b45] Elion G. B., Singer S. & Hitchings G. H. Antagonists of nucleic acid derivatives. VIII. Synergism in combinations of biochemically related antimetabolites. J. Biol. Chem. 208, 477–488 (1954). 13174557

[b46] Loregian A. . The 6-aminoquinolone WC5 inhibits human cytomegalovirus replication at an early stage by interfering with the transactivating activity of viral immediate-early 2 protein. Antimicrob. Agents Chemother. 54, 1930–1940 (2010). 20194695 10.1128/AAC.01730-09 PMC2863603

[b47] Luganini A. . Inhibition of herpes simplex virus type 1 and type 2 infections by peptide-derivatized dendrimers. Antimicrob. Agents Chemother. 55, 3231–3239 (2011). 21576438 10.1128/AAC.00149-11 PMC3122415

[b48] Prichard M. N. & Shipman C. Jr. A three-dimensional model to analyze drug-drug interactions. Antiviral Res. 14, 181–205 (1990). 2088205 10.1016/0166-3542(90)90001-n

[b49] Ensoli F. . HIV-1 Tat immunization restores immune homeostasis and attacks the HAART-resistant blood HIV-1 DNA: results of a randomized phase II exploratory clinical trial. Retrovirology. 12, 33 (2015). 25924841 10.1186/s12977-015-0151-y PMC4414440

[b50] Cavalli R. . Enhanced antiviral activity of acyclovir loaded into nanoparticles. Methods Enzymol. 509, 1e19 (2012). 22568898 10.1016/B978-0-12-391858-1.00001-0

[b51] Mulato A. S. & Cherrington J. M. Anti-HIV-1 activity of adefovir (PMEA) and PMPA in combination with antiretroviral compounds: *in vitro* analyses. Antiviral Res. 36, 91–97 (1997). 9443665 10.1016/s0166-3542(97)00043-0

[b52] Miller M. D. & Gibbs C. S. *In Vitro* Synergy of Tenofovir Combinations Against HIV-1. Gilead Sciences Report No. C1278-00005. March 6, (2001).

[b53] Cole A. L. . The retrocyclin analogue RC-101 prevents human immunodeficiency virus type 1 infection of a model human cervicovaginal tissue construct. Immunology. 121, 140–145 (2007). 17250585 10.1111/j.1365-2567.2006.02553.x PMC2265926

[b54] Rohan L. C. . *In vitro* and *ex vivo* testing of Tenofovir shows it is effective as an HIV-1 microbicide. PlosOne. 5, e9310 (2010). 10.1371/journal.pone.0009310 PMC2824823 20174579

[b55] Diomede L. . Passively transmitted gp41 antibodies in babies borm from subtype C HIV-1 seropositive women: correlation between finer specificity and protection. J Virol. 86, 4129–4138 (2012). 22301151 10.1128/JVI.06359-11 PMC3318605

[b56] Provine N. M., Cortez V., Chohan V. & Overbaugh J. The neutralization sensitivity of viruses representing human immunodeficiency virus type 1 variants of diverse subtypes from early in infection is dependent on producer cell, as well as characteristics of the specific antibody and envelope variant. Virology. 427, 25–33 (2012). 22369748 10.1016/j.virol.2012.02.001 PMC3321740

[b57] Provine N. M., Puryear W. B., Wu X., Overbaugh J. & Haigwood N. L. The infectious molecular clone and pseudotyped virus models of human immunodeficiency virus type 1 exhibit significant differences in virion composition with only moderate differences in infectivity and inhibition sensitivity. J. Virol. 83, 9002–9007 (2009). 19535443 10.1128/JVI.00423-09 PMC2738156

[b58] Kwong P. D. . Structure of an HIV-1 gp120 envelope glycoprotein in complex with the CD4 receptor and a neutralizing human antibody. Nature. 393, 648–659 (1998). 9641677 10.1038/31405 PMC5629912

[b59] Selhorst P. . M48U1 CD4 mimetic has a sustained inhibitory effect on cell-associated HIV-1 by attenuating virion infectivity through gp120 shedding. Retrovirology 10, 12 (2013). 23375046 10.1186/1742-4690-10-12 PMC3571899

[b60] Daar E. S., Li X. L., Moudgil T. & Ho D. D. High concentrations of recombinant soluble CD4 are required to neutralize primary human immunodeficiency virus type 1 isolates. Proc. Natl. Acad. Sci. USA 87, 6574–6578 (1990). 2395859 10.1073/pnas.87.17.6574 PMC54579

[b61] Sullivan N., Sun Y., Li J., Hofmann W. & Sodroski J. Replicative function and neutralization sensitivity of envelope glycoproteins from primary and T-cell line-passaged human immunodeficiency virus type 1 isolates. J. Virol. 69, 4413–4422 (1995). 7769703 10.1128/jvi.69.7.4413-4422.1995 PMC189183

[b62] Haim H. . Soluble CD4 and CD4-mimetic compounds inhibit HIV-1 infection by induction of a short-lived activated state. PLoS Pathog. 5, e1000360 (2009). 19343205 10.1371/journal.ppat.1000360 PMC2655723

[b63] Anderson D. J. . Targeting Trojan Horse leukocytes for HIV-1 prevention. AIDS. 24, 163–187 (2010). 20010071 10.1097/QAD.0b013e32833424c8 PMC4097178

[b64] Ariën K. K., Jespers V. & Vanham G. HIV-1 sexual transmission and microbicides. Rev. Med. Virol. 21, 110–133 (2011). 21412935 10.1002/rmv.684

[b65] Barditch-Crovo P. . Phase I/II trial of the pharmacokinetics, safety, and antiretroviral activity of tenofovir disoproxil fumarate in human immunodeficiency virus-infected adults. Antimicrob. Agents Chemother. 45, 2733–2739 (2001). 11557462 10.1128/AAC.45.10.2733-2739.2001 PMC90724

[b66] Van Rompay K. K. . Biological effects of short-term or prolonged administration of 9-[2-(phosphonomethoxy) propyl]adenine (tenofovir) to newborn and infant rhesus macaques. Antimicrob. Agents Chemother. 48, 1469–1487 (2004). 15105094 10.1128/AAC.48.5.1469-1487.2004 PMC400569

[b67] Tien D. . *In vitro* and *in vivo* characterization of a potential universal placebo designed for use in vaginal microbicide clinical trials. AIDS Res. Hum. Retrov. 10, 845–853 (2005). 10.1089/aid.2005.21.845 16225411

[b68] Andrei G. . Topical Tenofovir, a microbicide effective against HIV-1, inhibits Herpes Simplex Virus-2 replication. Cell Host Microb. 10, 379–389 (2011). 10.1016/j.chom.2011.08.015 PMC3201796 22018238

[b69] Grammen C., Augustijns P. & Brouwers J. *In vitro* profiling of the vaginal permeation potential of anti-HIV-1 microbicides and the influence of formulation excipients. Antiviral Res. 96, 226–233 (2012). 23000496 10.1016/j.antiviral.2012.09.011

[b70] Cates W. Jr After CAPRISA 004: time to re-evaluate the HIV-1 lexicon. Lancet 376, 495–496 (2010). 20709216 10.1016/S0140-6736(10)61200-7

[b71] Abu-Raddad L. J. . Genital herpes has played a more important role than any other sexually transmitted infection in driving HIV-1 prevalence in Africa. PLoS One. 3, e2230 (2008). 18493617 10.1371/journal.pone.0002230 PMC2377333

[b72] Wald A. & Link K. Risk of Human immunodeficiency virus infection in Herpes simplex type 2 sieropositive persons: a meta-analysis. J. Infect. Dis. 185, 45–52 (2002). 11756980 10.1086/338231

