# Supplementary material for: M48U1 and Tenofovir combination synergistically inhibits HIV infection in activated PBMCs and human cervicovaginal histocultures
Source: Sci Rep. 2017 Feb 1;7:41018. doi: 10.1038/srep41018 (PMC5286506; doi:10.1038/srep41018)
Supplement: Supplementary Figure 1 [file srep41018-s1.pdf]

M48U1 and Tenofovir combination synergistically inhibits HIV infection in activated PBMCs and human cervicovaginal histocultures

Giuseppina Musumeci, Isabella Bon, David Lembo, Valeria Cagno, Maria Carla Re, Caterina Signoretto, Erica Diani, Lucia Lopalco, Claudia Pastori, Loïc Martin, Gilles Ponchel, Davide Gibellini, Kawthar Bouchemal

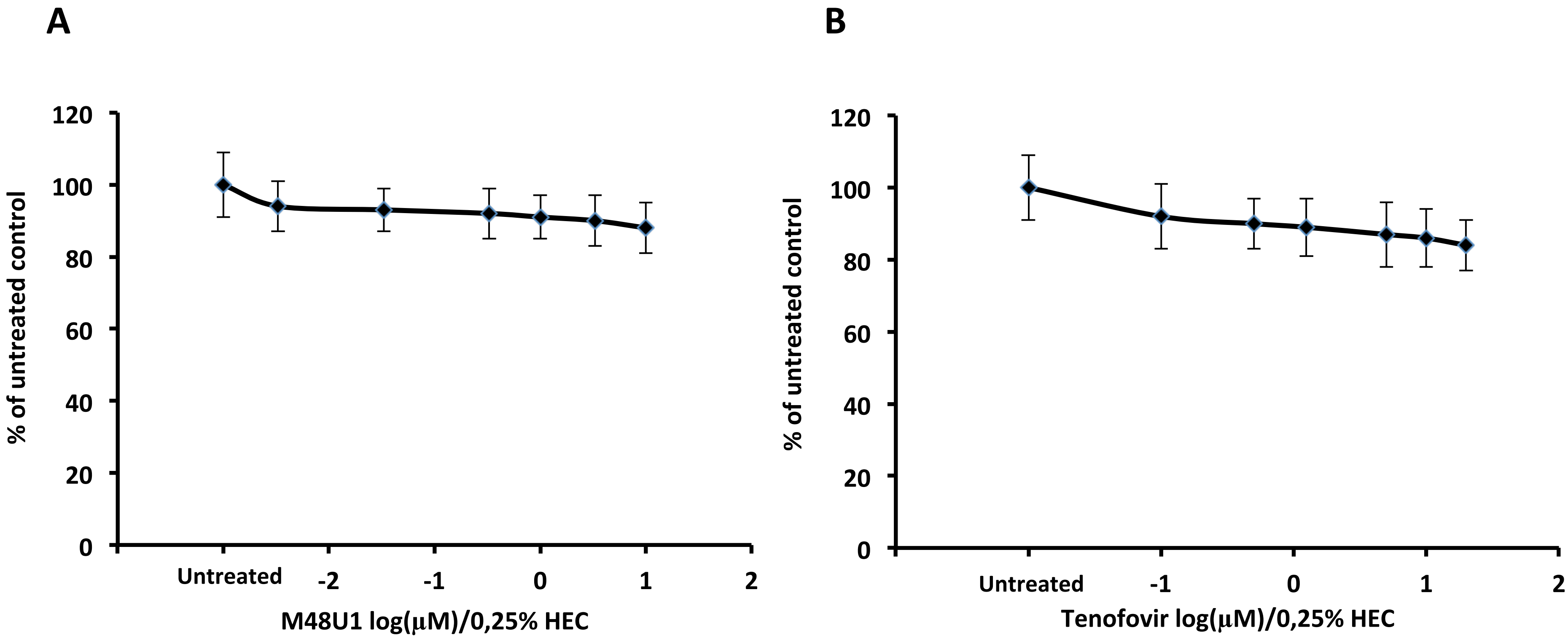

Supplementary figure 1. *M48U1 and tenofovir formulated with 0.25% HEC were not toxic to activated PBMCs*. The Trypan Blue technique was used to assess the viability of cells on day 7 post-infection. The data were expressed as the mean (±SD) of viable cells relative to untreated controls (set to 100%) obtained from three independent experiments performed in triplicate.
